# An acidic morpholine derivative containing glyceride from thraustochytrid, *Aurantiochytrium*

**DOI:** 10.1038/s41598-021-85636-1

**Published:** 2021-03-25

**Authors:** Kunimitsu Kaya, Fujio Shiraishi, Tetsuo Iida, Masaki Yamada, Tomoharu Sano

**Affiliations:** 1 Laboratory of Bioactive Compounds, Seaact Co. Ltd. TCI, 2-6-1, Sengen, Tsukuba, Ibaraki 305-0047 Japan; 2 Center for Environmental Measurement and Analysis, National Institute for Environmental Studies, 16-2 Onogawa, Tsukuba, Ibaraki 305-0053 Japan; 3 Global Application Development Center, Shimadzu Corporation, 1, Nishinokyo Kuwaharacho, Nakagyo-ku, Kyoto 604-8511 Japan

**Keywords:** Glycerides, Microbiology

## Abstract

A novel acidic morpholine-derivative containing glyceride (M-glyceride) was isolated from the cells of two strains of the thraustochytrid, *Aurantiochytrium*. The glyceride accounted for approximately 0.1 -0.4% of the lyophilized cells. The glyceride consisted of peaks I (85%) and II (15%). The structures of the intact and acetylated glycerides were elucidated by liquid chromatography-quadrupole time-of-flight chromatograph mass spectrometer (LC–Q/TOF) and NMR spectroscopy. The hydrate type of M-glyceride was detected as a minor component by LC–MS/MS. By 2D-NMR experiments, peaks I of the intact M-glyceride were elucidated as 1,2-didocosapentaenoyl-glyceryl-2′-oxy-3′-oxomorpholino propionic acid, and peak II was estimated 1,2-palmitoyldocosapentaenoyl- and/or 1,2-docosapentaenoylpalmitoyl-glyceryl-2′-oxy-3′-oxomorpholino propionic acid. The double bond position of docosapentaenoic acid was of the ω − 6 type (C_22_ = 5.ω − 6). The M-glyceride content varied by the cell cycle. The content was 0.4% of lyophilized cells at the mid logarithmic phase, and decreased to 0.1% at the mid stationary phase. When cells were grown in 1.0 µM M-glyceride-containing growth media, cell growth was stimulated to 110% of the control. With 0.1 µM acetyl M-glyceride, stimulation of 113% of the control was observed. Finding morpholine derivatives in biological components is rare, and 2-hydroxy-3-oxomorpholino propionic acid (auranic acid) is a novel morpholine derivative.

## Introduction

The thraustochytrid *Aurantiochytrium* has been examined extensively as a producer of high contents of squalene, docosahexaenoic acid (DHA) and pentadecanoic acids as an odd-carbon chain fatty acid^1–4^. It is well known that the major lipids of *Arantiochytrium* are comprised of squalene, carotenoids, triglyceride, glycolipids and phospholipids^5–8^.

During the investigation of acidic lipids from cells of *Aurantiochytrium* sp. SYLR6#3 and NB6-3, we found a novel acidic morpholine derivative containing glyceride (M-glyceride), which consisted of 2-hydroxy-3-oxo- morpholino propionic acid, glycerol and fatty acids.

Morpholine, a six-membered heterocyclic compound containing nitrogen and oxygen atoms, is an important moiety in many industrial and organic syntheses, and is often utilized in the field of medicinal chemistry for its advantageous physicochemical, biological, and metabolic properties^9^. Nevertheless, finding morpholine derivatives from biological samples is very rare.

In this paper we describe the chemical structural elucidation of M-glyceride isolated from *Aurantiochytrium* cells and their cell growth activity.

## Results

### Fractionation and purification of M-glyceride

Cells were harvested 72 h after inoculation, and the yield of M-glyceride was obtained as approximately 0.2% of the lyophilized cells. M-glyceride showed an R*f* 0.73 on TLC using chloroform/methanol/water (5:5:1, v/v/v) as the solvent. The spot on the TLC plate was negative against the molybdenum reagent^10^.

**Figure 1:**
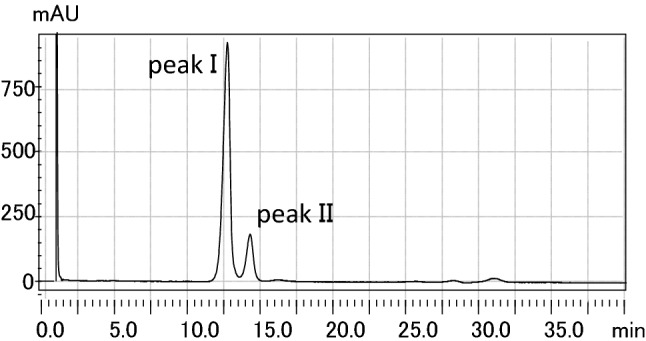
HPLC chromatogram of M-glyceride. M-glyceride was detected by HPLC with a photodiode array detector (PDA) and a Wakosil 5NH2 column (2.0 × 150 mm). Eluted compounds were detected at 205 nm.

On the HPLC chromatogram of purified M-glyceride, two peaks were detected at Rt-12.54 (peak I) and 14.48 min (peak II). Peaks I and II comprised 85% and 15% of the total area, respectively (Fig. 1). M-glyceride was found in *Aurantiochytrium* sp, SYLR6#3 and NB6-3.

### Analysis of NMR and MS spectra of M-glyceride

The ^1^H and ^13^C-NMRchemical shifts for peak I of M-glyceride are shown in Table 1. Two-dimensional spectra, ^1^H–^1^H-COSY, DEPT, HSQC and HMBC, were obtained. From these spectra, the existence of glycerol was found. The protons were assigned as H-1 (2H, δ, 4.18 ppm), H-2 (1H, d, 5.26 ppm) and H-3 (2H, δ, 3.73 and ppm). Furthermore, carbons at positons 1 and 2 of glycerol were esterified with fatty acids. The C-3 of glycerol was combined with a polar moiety by an ether linkage. In the ^13^C NMR spectrum, four quaternary carbons were detected between 160 and 180 ppm. The HMBC spectra suggested that the carbons at C-1 of DPA I (173.38 ppm) and DPA II (173.67 ppm) were assigned as esterified. The chemical shift at 177.17 ppm suggested that a carboxylic acid group was present in M-glyceride. The quaternary carbon at 169.34 ppm was suggested to be the carbonyl carbon at C-3 (> C=O) of the morpholine ring.

**Table 1:** ^1^H-NMR and ^13^C-NMR data for M-glyceride.

Position	1H	*J* (Hz)	13C	HMBC (^1^H→^13^C)
**Glycerol (G)**				
1	4.18	(dd, 3.0, 7.8)	63.44	DPA-I-C1
	4.33	(dd,3.0, 7.8)		
2	5.26	(m)	70.93	
3	3.73	(m)	65.47	M-C2
	4.12	(m)		
**DPA-I**				
1			173.38	
2	2.29	(m)	29.31	
3	1.54	(m)	25.12	
4–20				
	2.07	(m)	27.61	
	2.85	(m)	25.99	
	5.38	(m)	128.38	
ω − 1	1.27	(m)	22.97	
ω	0.89	(m)	14.24	
**DPA-II**				
1			173.67	
2	2.37	(m)	29.32	
3	1.58	(m)	27.21	
4–20				
	2.07	(m)	27.61	
	2.85	(m)	25.99	
	5.38	(m)	128.38	
ω − 1	1.27	(m)	23.00	
ω	0.97	(m)	14.54	
**Morpholine (M)**				
1				
2	4.80	s	100.66	M-C3
3			169.34	
4				
5	3.16	(dt, 3.8, 10.8)	45.41	M-C3
	3.62	(m)		
6	3.75	(m)	65.47	M-C2
	3.83	(m)		
**Propionic acid (P)**				
1			176.97	
2	2.40	(m)	34.38	P-C1
3	3.19	(dt, 6.3, 13.2)	47.50	P-C1, M-C3, M-C5
	3.46	(m)		

M-glyceride: 1,2-di-docosapentaenoylglyceryl-2′-oxy-3′-oxomorpholino propionic acid.

The mixture of CDCl_3_ and CD_3_OD (1: 1,v/v) was used as a solvent.

Multiplets in the table are due to overlapped peaks, except G-2.

*DPA* docosapentaenoic acid, *dd* doublet of doublet, *s* singlet, *dt* doublet of triplet, *m* multiplet.

From the NMR results, the structure of peak I of M-glyceride was determined as a diacylglyceride combined with a morpholine moiety (C_7_H_10_NO_5_) by an ether linkage.

In negative ion mode of the MS spectra, values of *m/z* 904.5948 [C_54_H_82_NO_10_. (calcd, 904.5944, *Δ* 0.4 mDa)] from peak I and *m/z* 830.5789, [C_48_H_80_NO_10_ (calcd, 830.5788, *Δ* 0.1 mDa)] from peak II were observed. From the major peak I, a fragment ion at *m/z* 574 [m/z 904–330 (C_22_H_34_O_2_)] was found. The liberated ion was due to the neutral loss of docosapentaenoic acid (C_22_ = 5, DPA). The other fragment ions were observed at *m/z* 886 [loss of H_2_O from *m/z* 904], 329 [C_22_H_34_O_2_–H (DPA)]^−^, 244 [C_10_H_15_NO_6_–H]^−^ and *m/z* 188 [C_7_H_10_NO_5_–H]^−^. In the case of peak II, fragment ions were observed at *m/z* 812 [loss of H_2_O from *m/z* 830], 574 [*m/z* 830–256 (C_16_H_32_O_2_)], 500 [*m/z* 830–330 (C_22_H_34_O_2_)], 329 [(C_22_H_34_O_2_)–H]^−^, and 255 [(C_16_H_32_O_2_)–H]^−^, *m/z* 244 [C_10_H_15_NO_6_–H]^−^ and 188 [C_7_H_10_NO_5_–H]^−^ were also detected. The fragment ion at *m/z* 255 was estimated as palmitic acid (C_16_ = 0). The fragment ions at *m/z* 244 [[C_10_H_15_NO_6_–H]^−^, 188 [C_7_H_11_NO_5_–H]^−^ and 170 [C_7_H_9_NO_4_–H]^−^ were found in the spectra of both peaks I and II. The results suggested that peaks I and II consisted of the same moiety. These results are summarized in Table 2.

**Table 2:** Negative mode MS/MS fragments of Peak I and II of M-glyceride.

Fragment formula	*m/z* Theoretical	*m/z* Observed	Error mmDa	Notes
**Peak I**				
C_54_H_82_NO_10_	904.5939	904.5948	0.9	[M·hydrate–H]^−^
C_54_H_80_NO_9_	886.5833	886.5829	0.4	[M–H]^−^
C_32_H_48_NO_8_	574.3380	574.3367	1.3	[M hydrate–330-H]^−^
C_22_H_33_O_2_	329.2481	329.2482	0.1	C_22_ = 5
C_10_H_14_NO_6_	244.0821	244.0818	0.3	
C_7_H_10_NO_5_	188.0559	188.0559	0	
C_7_H_8_NO_4_	170.0453	170.0456	0.3	
**Peak II**				
C_48_H_80_NO_10_	830.5782	830.5789	0.7	[M·hydrate–H]^−^
C_48_H_78_NO_9_	812.5677	812.5670	0.7	[M–H]^−^
C_32_H_48_NO_8_	574.3380	574.3396	1.6	[M hydrate–256-H]^−^
C_26_H_46_NO_8_	500.3223	500.3227	0.4	[M hydrate–330-H]^−^
C_22_H_33_O_2_	329.2481	329.2488	0.7	C_22_ = 5
C_16_H_31_O_2_	255.2324	255.2323	0.1	C_16_ = 0
C_10_H_14_NO_6_	244.0821	244.0818	0.3	
C_7_H_10_NO_5_	188.0559	188.0560	0.1	
C_7_H_8_NO_4_	170.0453	170.0461	0.8	

M-glyceride: 1,2-diacylglyceryl-2′-oxy-3′-oxomorpholino propionic acid.

C_22_ = 5, docosapentaenoic acid (DPA); C_16_ = 0, palmitic acid.

The formula of the morpholine moiety obtained from MS was different from that (C_7_H_10_NO_5_) obtained from NMR. The MS results suggested that the morpholine moiety was C_7_H_12_NO_6_. The MS data of the morpholine moiety are explained as [C_7_H_10_NO_5_ + H_2_O]. This suggests that an extra H_2_O combined somewhere with the moiety and it exists as a hydrate. From the NMR and MS results, we considered that peaks I and II contained M-glyceride as the major component and its hydrate as the minor component (Table 2). The proton and related quaternary carbon signals of the hydrate were too weak for NMR analysis. However, assignment of the morpholine moiety was still unclear, since many ^1^H-NMR signals due to fatty acid moieties overlapped with those of the morpholine moiety.

### Elucidation of the acetylated M-glyceride

**Figure 2:**
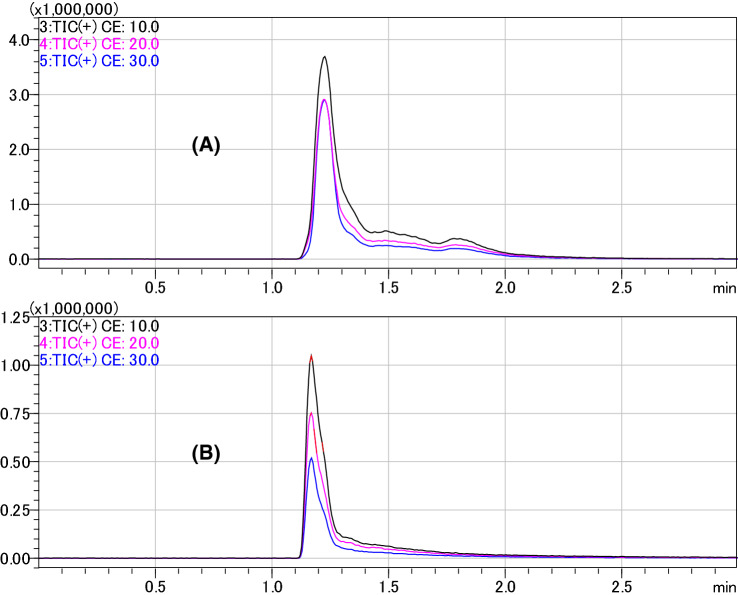
Total ion chromatograms of the product ion of acetylated M-glyceride (**A**) and acetylated M-glyceride-hydrate (**B**) by the LC–MS/MS. Precursor ions *m/z* 348.15 for acetylated M-glyceride [C_14_H_21_NO_9_ + H]^+^ and *m/z* 366.15 for acetylated M-glyceride hydrate [C_14_H_23_NO_10_ + H]^+^ were applied to LC–MS/MS (Q-TOF) in positive mode for the total ion chromatograms of the product ion. The retention times of acetylated M-glyceride (**A**) and acetylated M-glyceride-hydrate (**B**) were 1.225 and 1.152 min, respectively. MS/MS product ion scan: *m/z* 10–1100. *CE* collision energy.

To elucidate the structure of the morpholine moiety, the fatty acids in M-glyceride were substituted with an acetyl group. Acetylated M-glyceride was analysed by reverse-phase LC–Q/TOF. When the precursor ion of *m/z* 348 (C_14_H_21_NO_9_) was applied to the LC–MS/MS, a large peak in the ion chromatogram of the total product ion was observed at Rt 1.225 min. In the case of the precursor ion of *m/z* 366 (C_14_H_23_NO_10_), a small broad peak was observed at Rt 1.152 min (Fig. 2). These results showed that M-glyceride comprised C14H21NO9 (M-glyceride) as the major peak (86%) and C_14_H_23_NO_10_ (M-glyceride hydrate) as the minor peak (14%).

The LC–MS/MS fragments in positive and negative mode of both glycerides are shown in Table 3. The greater part of the exact m/z of the fragments of acetylated M-glyceride and its hydrate obtained from both modes agreed well with each other. These results suggested that the structures of both the glyceride and hydrate agreed.

**Table 3:** Positive and negative modes MS/MS fragments for acetylated M-glyceride and acetylated M-glyceride·hydrate.

Positive ion mode [M + H]^+^ (*m/z*)		Negative ion mode [M − H]^−^ (*m/z*)		(M)	Formula
M-glyceride	M-glyceride·hydrate	M-glyceride	M-glyceride hydrate		
	366.1392		364.1244	(M, 365)	C_14_H_23_NO_10_
348.1293	348.1288	346.1132	346.1169	(M, 347)	C_14_H_21_NO_9_
330.1179	330.1182			(M, 329)	C_14_H_19_NO_8_
306.1182	306.1181	304.1033	304.1038	(M, 305)	C_12_H_19_NO_8_
			292.1036		
288.1083	288.1083			(M, 287)	C_12_H_17_NO_7_
		262.0935	262.0916	(M, 263)	C_10_H_17_NO_7_
270.0969					
246.0969	246.0967	244.0839	244.0836	(M, 245)	C_10_H_15_NO_6_
		232.0824	232.0827	(M, 233)	C_9_H_15_NO_6_
232.0811	232.0814			(M, 231)	C_9_H_13_NO_6_
228.0864					
214.0685			212.0578	(M, 213)	C_9_H_11_NO_5_
		190.0721	190.0715	(M, 191)	C_7_H_13_NO_5_
190.0714	190.0708			(M, 189)	C_7_H_11_NO_5_
	176.0913		174.0769	(M, 175)	C_7_H_13_NO_4_
172.0601	172.0598	170.0455	170.0452	(M, 171)	C_7_H_9_NO_4_
		158.0465	158.0461	(M, 159)	C_7_H_11_NO_3_
159.0655					
	158.0808				

The formula of the fragments showed that all structures of the fragments included a morpholino propionic acid moiety.

As shown in Tables 2 and 3, the fragment formulas of C_10_H_15_NO_6_ (*m/z* 244 in negative mode and *m/z* 246 in positive mode), C_7_H_11_NO_5_ (*m/z* 188 in negative mode), and C_7_H_9_NO_4_ (*m/z* 170 in negative mode and *m/z* 172 in positive mode) were obtained from both the intact M-glyceride and acetylated M-glyceride. Therefore, the results showed that the morpholino propionic acid moiety in the intact M-glyceride was preserved in acetylated M-glyceride.

Extensive NMR analysis by ^1^H–^1^H COSY and HMBC spectroscopy revealed the spin systems of glycerol, morpholine and propionic acid (Table 4). The presence of two acetyl groups supported that the intact glyceride was diacyl glyceride. On the morpholine and glycerol units, the H-2 (δ 4.84 ppm) of morpholine suggested an ether linkage between C-2 of morpholine and C-3 of glycerol. The correlation of the HMBC spectra also supported this linkage.

**Table 4:** ^1^H-NMR and ^13^C-NMR data for acetylated M-glyceride.

Position	1H	*J* (Hz)	13C	HMBC (^1^H→^13^C)
**Glycerol (G)**				
1	4.18	(d, 3.0)	62.66	Ac-I-C1
	4.33	(d, 3.0)		
2	5.22	(m)	70.11	
3	3.70	(m)	57.11	M-C2
	4.08	(m)		
**Acetyl-I (Ac-I)**				
1	4.12	(m)	170.42	
2	2.04	(s)	20.75	
**Acetyl-II (Ac-II)**				
1			170.69	
2	2.06	(s)	20.86	
**Morpholine (M)**				
1				
2	4.84	(s)	96.22	M-C3
3			163.91	
4				
5	3.18	(dt,3.8, 10.8)	46.16	M-C3
	3.62	(m)		
6	3.75	(m)	66.91	M-C2
	3.83	(m)		
**Propinic acid (P)**				
1			177.42	
2	2.45	(m)	33.60	P-C1
3	3.48	(dt, 6.3,13.2)	43.74	P-C1, M-C3, M-C5
	3.70	(m)		

Acetyl M-glyceride: 1,2-di-acetylglyceryl-2′-oxy-3′-oxomorpholino propionic acid.

CDCl_3_ was used as a solvent.

Multiplets in Table 4 are due to overlapped peaks except G-2.

*dd* doublet of doublet, *s* singlet, *dt* doublet of triplet, *m* multiplet.

The structure of 2-oxy-3-oxomorpholino propionic acid was deduced from the COSY and HMBC spectra. In the COSY spectra, the relation of connectivity from H-5 (δ 3.18 ppm) to H-6 (δ 3.75 ppm) of the morpholine unit was determined. On the morpholine ring unit, the chemical shift of H-6 (δ 3.75 ppm) and C-6 (δ 66.91 ppm) suggested that C-6 was connected to an oxygen atom. Additionally, the chemical shifts of H-5 (δ 3.18 ppm) and C-5 (δ 46.16 ppm) suggested that C-5 was connected to a nitrogen atom. Furthermore C-3 of the morpholine ring unit correlated with H-2 and H-5 of the ring in the HMBC spectrum. An HMBC correlation was also observed between C-2 and H-6 of the morpholine ring. From these results, the morpholine ring was elucidated.

On the propionic acid unit of the morpholine moiety, the relation of the connectivity from H-2 (δ 2.45 ppm) to H-3 (δ 3.48 ppm) was observed in the COSY spectrum, and the correlations of C-1 (δ 177.42 ppm) with H-2 and H-3 of the propionic acid unit were found in the HMBC spectrum. The HMBC spectrum also showed that H-3 of the propionic acid unit correlated with C-3 and C-5 of the morpholine ring unit.

**Figure 3:**
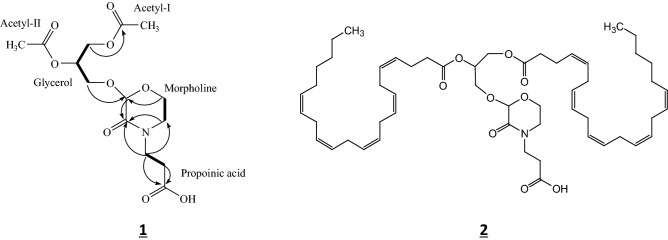
HMBC (^1^H→^13^C, arrow) and COSY (heavy line) correlation of acetylated M-glyceride (1,2-di-acetylglyceryl-2′- oxy-3′-oxomorpholino propionic acid) (**1**) and structure of the major intact M-glyceride (1,2-di-docosapentaenoylglyceryl-2′-oxy-3′-oxo-morpholino propionic acid) (**2**).

From these results, the structure of acetylated M-glyceride was elucidated as 1,2-diacetylglyceryl-2’-oxy-3’-oxomorpholino propionic acid (Fig. 3, 1), whereas the structure of the extra H_2_O containing acetylated M-glyceride (hydrate) was still unclear.

### Elucidation of the intact M-glyceride

The above structure was confirmed by comparison of the MS/MS fragmentation of the polar moieties of the intact M-glyceride and its acetylated derivative. From the above results, *m/z* 904 of peak I in Table 2 was shown as {[M-glyceride hydrate–H]^−^ along with the *m/z* of 830 of peak II.

The fragment ions at *m/z* 244 [C_10_H_15_O_6_–H]^−^, 188 [C_7_H_11_NO_5_–H]^−^ and 170 [C_7_H_9_NO_4_–H]^−^ were observed in both the MS/MS spectra of the intact M-glyceride and its acetylated derivative. Furthermore, HMBC correlations on the morpholine ring of the intact M-glyceride were the same as that of the acetylated M-glyceride.

The intact M-glyceride consisted of two fatty acid molecules. Peak I, as the major peak (Fig. 1) contained two molecules of DPA, and the minor peak (peak II) contained DPA and palmitic acid (C_16_ = 0).

Regarding the chemical shifts of DPA in peak I of the intact glyceride in the ^1^H- and ^13^C-NMR spectra (Table 2), C-1 of DPA-I and DPA II were observed at δ, 173.38, and 173.67 ppm, respectively. H-2 of DPA-I (δ 2.29 ppm) and DPA II (δ 2.37 ppm), H-3 of DPA-I (δ 1.54 ppm) and DPA-II (δ 1.58 ppm) and H- 22 as the terminal of the carbon chain of DPA-I (δ 0.89 ppm) and DPA-II (δ, 0.97 ppm) were assigned. Double-bond related signals of DPA, –CH2-C**H**_**2**_-CH = (δ 2.07 ppm), = CH-C**H**_**2**_-CH = (δ 2.85 ppm) and –C**H** = C**H**-(δ 5.38 ppm), were also assigned.

DPA has two isomers of the ω − 3 and ω − 6 types. To confirm the isomers, the fatty acids of peaks I and II were oxidized using KMnO4/KIO_4_^11^, and the formed alkanoic acid was identified as a methyl ester derivative by GC. A peak was detected at Rt 4.763 min. This Rt agreed well with authentic methyl hexanoate. From these results, DPA was identified as ω − 6 docosapentaenoic acid. The fatty acid of peak II was also identified as ω − 6.

From the above results, the major M-glyceride peak I was elucidated as 1,2-didocosapentaenoyl-(C_22_ = 5, ω − 6) glyceryl-2′-oxy-3′-oxomorpholino propionic acid (Fig. 3, 2), and the minor one was estimated as 1,2-palmitoyl-docosapentaenoyl- and/or 1,2-docosapentaenoyl-palmitoyl-gryceryl-2′-oxy-3′-oxomorpholino propionic acid.

### Changes in the M-glyceride content in the cell cycle of *Aurantiochytrium* sp. SYLR6#3

The content of M-glyceride during cultivation of *A*. sp. SYLR6#3 was examined and was found to vary (Table 5). The highest content (0.4% of the lyophilized cells) was obtained from cells at the mid logarithmic phase. In cells at the end of the logarithmic (or early stationary) phase, the M-glyceride content was approximatelly 0.2%, which gradually decreased and showed a constant value until the death phase. In cells at the mid stationary and death phases, the value was approximately 0.1%. The variation in M-glyceride content in the life cycle of cells seems to be related to cell growth.

**Table 5:** Changes in M-glyceride contents in cells of *Aurantiochytrium* sp. SYLR6#3 during culture.

Day after inoculation	Day 1	Day 2	Day 4	Day 7
Cell growth phase	Logarithmic	Early stationary	Mid stationary	Death
Lyophilized cell weight (g/L)	2.6 ± 0.1	4.2 ± 0.1	4.5 ± 0.2	3.4 ± 0.2
M-glyceride content (mg/g lyophilized cells)	3.99 ± 0.14	2.33 ± 0.08	1.06 ± 0.06	1.16 ± 0.06

Biomass was expressed as g/L of lyophilized cells with ± SD (n = 4).

M-glyceride contents were expressed as mg/g of lyophilized cells with ± SD (n = 4).

M-glyceride content was determined by HPLC with PDA detector (205 nm) and Wakosil 5NH2 (2.0 × 150 mm) column.

### Effect of M-glyceride on cell growth

To examine the cell growth activity of M-glyceride, peak I of M-glyceride was isolated by HPLC. M-glyceride was composed of 86% di-DPA M-glyceride and 14% of its hydrate. Additionally, acetylated M-glyceride was comprised of the same ratio as that of M-glyceride.

*Aurantiochytrium* sp. SYLR6#3 cells were cultured with various concentrations of M-glyceride or acetylated M-glyceride containing growth medium in 16-well plates at 25 °C for 24 or 48 h. M-glyceride was easily emulsified in the medium, whereas acetylated M-glyceride dissolved well in the medium. In the case of M-glyceride, cell growth was only stimulated significantly at the concentration of 1.0 µM during 48 h of cultivation (Table 6). For 24 h of cultivation, cell growth was inhibited at concentrations of 100 and 1000 µM. When acetylated M-glyceride was examined, cell growth was only stimulated significantly at a concentration of 0.1 µM for 48 h of cultivation. Acetylated M-glyceride seems to be more effective for the cell growth stimulation than M-glyceride. Most likely, the stimulation effect was related to the solubility of the acetylated M-glyceride.

**Table 6:** Effect of M-glyceride and acetyl M-glyceride on cell growth of *Aurantiochytrium* sp. SYLR6#3.

	Cell concentration (absorbance at 650 nm)		
	0 h^a^	24 h^a^	48 h^a^
**Conc. of M-glyceride (µM)**			
0 (control)	0.81 ± 0 (100)	4.2 ± 0.13 (100)	6.2 ± 0.08 (100)
0.1	0.81 ± 0 (100)	4.2 ± 0.18 (100)	6.3 ± 0.24 (102)
1.0	0.81 ± 0 (100)	4.3 ± 0.24 (102)	6.8 ± 0.21** (110)
10.0	0.81 ± 0 (100)	3.9 ± 0.21 (93)	6.4 ± 0.29 (103)
100	0.81 ± 0 (100)	3.6 ± 0.10* (86)	5.9 ± 0.22 (95)
1000	0.81 ± 0 (100)	3.7 ± 0.05* (88)	5.6 ± 0.19* (90)
**Conc. of acetylated M-glyceride (µM)**			
0 (control)	0.82 ± 0 (100)	4.1 ± 0.10 (100)	6.1 ± 0.26 (100)
0.01	0.82 ± 0 (100)	4.1 ± 0.12 (100)	6.3 ± 0.21 (103)
0.1	0.82 ± 0 (100)	4.0 ± 0.06 (98)	6.9 ± 0.25** (113)
1.0	0.82 ± 0 (100)	4.1 ± 0.17 (100)	6.4 ± 0.10 (105)
10	0.82 ± 0 (100)	4.0 ± 0.21 (98)	6.4 ± 0.14 (105)
100	0.82 ± 0 (100)	3.8 ± 0.20 (93)	6.1 ± 0.28 (100)

Cells of *A*. sp. were cultured with M-glyceride or acetylated M-glyceride containing a growth medium using 16 well plates at 25 °C for 24 or 48 h. Cell concentrations were determined using a photoelectric colorimeter at 650 nm, and were expressed as absorbance of cell suspension.

Values are averages of independent experiments with ± SD (n = 4).

M-glyceride: 1,2- didocosapeentaenylglyceryl-2′-oxy-3′-oxomorpholino propionic acid; Acetylated M-glyceride: 1.2 diacetylglyceryl-2′-oxy-3′-oxomorpholino propionic acid; M-glyceride and acetyl M-glyceride were included 14% of their hydrate type.

Differences of cell concentrations after inoculation between control and the M-glyceride or acetylated M-glyceride containing media were tested statistically significant by Student’s *t* test.

^a^Time (h) after inoculation.

**Cell growth stimulation *p* < 0.005; *cell growth inhibition *p* < 0.005.

## Discussion

### M-glyceride hydrate

The molecular formula (C_14_H_23_NO_10_) of acetylated M-glyceride hydrate was obtained from MS/MS spectra (Table 3), whereas the molecular formula of acetylated M-glyceride obtained from ^1^H-NMR and ^13^C-NMR was C_14_H_21_NO_9_. The extra H_2_O was found in the formulae from MS/MS. Most likely, the extra H_2_O combined with the morpholine ring as a hydrate. If the H_2_O formed a ketone hydrate, the ^13^C-NMR chemical shift of [> C(OH)2] ought to have appeared at 100 ppm, as shown for the chemical shift of C-2 [> C(–O–)2, 96.22 ppm] of the morpholine moiety. However, the chemical shift of C-3 appeared at 163.91 ppm (Table 4). This chemical shift suggested that C-3 was not a ketone hydrate but rather aketone (> C=O). Furthermore, the existence of ketone hydratse in amide linkages is not known.

In the present report, we could not assign the position of the hydrate in M-glyceride. Most likely, the hydrate type in the M-glyceride fraction was dehydrated by the ionization energy of MS/MS. The MS/MS spectra of the acetylated M-glyceride and its hydrate showed that the M-glyceride hydrate was converted to M-glyceride by dehydration.

#### Changes in M-glyceride content and cell growth

The changes in M-glyceride content were observed. The highest content was observed in cells in the logarithmic phase (24 h after inoculation) of the mitotic period. M-glyceride may play an important role in cell division. Most likely, the concentration of endogenous M-glyceride is sufficient for cell division, and exogenous M-glyceride is not necessary. After the logarithmic phase, the concentration of endogenous M-glyceride rapidly decreased, and cell division slows down. At this time, exogenous M-glyceride may be incorporated and utilized for cell growth.

M-glyceride is a negative charged biosurfactant. At a high concentration (100 or 1000 µM), cell growth was inhibited after 24 h of cultivation. This inhibition may be related to the surfactant action of M-glyceride, because the inhibition induced by acetylated M-glyceride was not observed clearly.

### Morpholine derivatives in natural products

A few reports on morpholine derivatives from natural products have been described. Chelonins were isolated from a marine sponge that had antimicrobial and anti-inflammatory activities^12^. Polygonapholine, a novel alkaloid, was isolated from *Polygonatum altelobatum*^13^. Furthermore, an anti-tumor antibiotic morpholine derivative was isolated from *Streptomyces globisporus*^14^. A morpholine derivative, *syn*-3-isopropyl-6-(4-methoxybenzyl)-4-methylmorpholine-2,5-dione, was isolated from the Thai Sea hare, *Bursatella leachii*^15^. The above findings on morpholine moieties in secondary metabolites have been considered rare cases. Our finding is unique because the morpholine moiety is a component of diacylglyceride as a lipid metabolite. The elucidated polar moiety, 2-hydroxy-3-oxomorpholino propionic acid (auranic acid) is a novel morpholine derivative.

The morpholine derivative -containing glyceride possibly plays an important role in the life cycle of *Aurantiochytrium* and other organisms.

Morpholine derivatives have been synthesized for medicinal drugs and their biological activities have been examined^9,16–19^. Morpholine derivatives isolated from natural products as in this study, may contribute to the development of a new medicinal drug.

## Methods

### Materials

DEAE- Sephadex-A25 was obtained from GE Healthcare UK Ltd (Amersham Place, Little Chalfont, Buckinghamshire HP7 9NA, England). Yeast extract and tryptone were purchased from Becton, Dickinson and Company (Sparks, MD, USA). Sea salt was obtained from Red Sea Salt USA (Huston, TX, USA). All other chemicals and solvents were of analytical grade.

### Microorganisms

*Aurantiochytrium* sp SYLR6#3 and NB6-3 isolated from seawater in the Okinawa Islands, Japan, were purchased from OP BIO Co. (Okinawa, Japan).

### Methods

#### Culture conditions

The seed culture medium contained 1% tryptone, 0.2% yeast extract and 2% glucose, which were dissolved in 1.2% sea salt solution.

Mass-culture was performed in 5 L air-lift bioreactors (with an initial 3.0 L of culture medium) equipped with a pH controller. Mixing of the medium was performed by air bubbling. The bioreactors were set up in a clean booth, which were maintained at 25 ± 1.2 °C. The mass-culture medium contained 3.6% glucose, 0.5% sodium glutamate, 1.0% tryptone, 0.2% yeast extract and 1.0% sea salt. Air was supplied at 1.2 vvm. To make bubbles, air was passed through a ceramic sparger. Cells were grown in the medium at pH 7.4, controlled by the addition of 1.0 M NaOH, and were harvested at 72 h after inoculation. For the 72h culture, the glucose content in the medium reached below 0.2%. Harvested cells were lyophilized and stored at − 25 °C.

#### Extraction and isolation of M-glyceride

Lipids were extracted with chloroform (C)/methanol (M) 2:1(v/v) from 10 g of lyophilized cell materials. To the extract, 0.2 volumes of 0.9% NaCl solution was added. After centrifugation, the lower phase was separated and evaporated using a rotary evaporator under reduced pressure. The remaining residue was dissolved with C, and fractionated by silica gel chromatography (bed volume: 20 mL, prepared with C), eluted with 100 mL of C, CM (9:1, v/v) and CM (1:4). The phospholipid (PL) fraction was recovered from CM (1:4). After removing the solvent, the PL fraction was resuspended in C/M/ water (W) (5:5:1, v/v/v). The suspension was applied to DEAE-Sephadex-25 (20 mL, formate-type), and 100 mL of C/M/W (5:5:1, v/v/v) was passed through the column, followed by 100 mL of C/M/0.2 M ammonium formate (5:5:1, v/v/v). The acidic PL fraction was eluted with an ammonium salt containing solvent. The eluate was evaporated under reduced pressure. The M-glyceride and acidic PL containing residue was suspended in 0.1% phosphoric acid containing water. M-glyceride was extracted from the suspension with n-hexane.

#### Thin layer chromatography (TLC)

To purify M-glyceride from the extract, the extract was applied to TLC silica gel plates containing a fluorescent indicator and developed with C/M/W (5:5:1, v/v/v) as the developing solvent. M-glyceride migrated at an R*f* of 0.73. Under UV light, the M-glyceride band was scraped off the plates and eluted with C/M (1:4, v/v). M-glyceride was re-chromatographed on silica gel plates using the same solvent system. The purified M-glyceride was utilized for further structural analysis.

#### Acetylation of M-glyceride

M-glyceride was subjected to mild alkaline hydrolysis at 25 °C for 1 h in methanolic 1.5 M NaOH^20^. After hydrolysis, the pH of the hydrolysate was adjusted to 4.0 with 1.5 M HCl. The fatty acids liberated from M-glyceride were extracted with n-hexane. After neutralization with 1.0 M NaOH, the aqueous phase was evaporated to dryness under a stream of N2. The remaining residue was subjected to acetylation using pyridine/acetic anhydride (1:4, v/v) at 25 °C overnight. After the reaction, the solution was dried under a stream of N2. The remaining residue was suspended in n-hexane, spotted on TLC plates and developed with CMW (5:5:1, v/v/v) as the solvent. The acetylated M-glyceride on TLC was detected by charring after spraying with 20% H2SO4. The R*f* value of the acetylated lipid was found to be 0.61. The acetylated M-glyceride was scraped off from the plates and extracted with CM (1:4, v/v). As a result, a colourless pasty material was obtained after drying under a stream of N2, which was utilized for NMR and LC–MS/MS analyses.

#### HPLC analysis of M- glyceride

M-glyceride was further purified using Shimadzu Prominence HPLC consisting of pumps (LC-20AD), a controller, a degasser, a column oven, a PDA detector and a workstation. The HPLC conditions were as follows: column, Wakosil 5 NH4 column (4.6 × 150 mm); column temperature, 40 °C; mobile phase, acetonitrile/methanol/0.2% trimethylamine in water adjusted to pH 4.0 with phosphoric acid (670 / 220 / 110, v/v/v); and flow rate, 1.0 mL/min. Peaks were detected at 205 nm.

#### GC analysis of fatty acids

Fatty acids of M-glyceride were converted to methyl esters using 14% BF_3_-methanol at 70 °C for 20 min. Fatty acid methyl esters were analysed using GC-FID (Shimadzu GC-2025) with a DB-23 column (60 m × 0.25 mm i.d., film thickness 0.15 µm; J&W Scientific). The GC operating conditions were as follows: column temperature, 50 °C held for 1 min, increased to 175 °C (at a rate of 25 °C /min), then increased to 230 °C (at a rate of 4 °C/min), and held for 5 min; FID port temperature, 250 °C; carrier gas (He) flow rate, 2.06 mL/min; FID hydrogen gas flow rate, 40 mL/min; and air flow rate, 450 mL/min.

#### NMR

^1^H NMR (500 MHz) and ^13^C NMR (125 MHz) spectra were recorded using a JEOL JNM-ECA500 spectrometer. CDCl3 or CDCl3/CD3OD (1:1, v/v) was used as the solvent.

#### LC–MS/MS

MS and MS/MS spectra were recorded on an LC/Q-TOF system consisting of a *Nexera* UHPLC and aquadrupole time-of-flight mass spectrometer (LCMS-9030,Shimadzu Corporation, Kyoto, Japan). LC conditions were as follows; column, Kinetex C8 (2.1 mmI.D. × 150 mm, 2.6 µm) Phenomenex, USA; solvent, isocratic, 30% of 20 mM ammonium formate-containing and 70% acetonitrile/isopropanol (1:1, v/v); flow rate, 0.4 mL/min; and column temperature, 40 °C. MS conditions were as follows: ionization mode, ESI positive/negative; capillary voltage, 4.5 kV (positive)/− 3.5 kV (negative); drying gas, 10 L/min; nebulizer gas, 2.0 L/min, heating gas, 10 L/min; interface temperature, 300 °C.

#### Cell growth activity

Cells of *A*. sp. were cultured with growth medium (1% tryptone, 0.2% yeast extract, 2% glucose, and 1.2% sea salt) containing various concentrations (1.0–1000 µM) M-glyceride or (0.1–100 µM) acetylated M-glyceride in 16-well plates at 25 °C for 24 or 48 h. Cell concentrations were determined using the photoelectric colourimeter at 650 nm and were expressed as the absorbance of the cell suspension.

## Data Availability

This analytical data were validated by HPLC. As shown in Table 5, the determination data were obtained by HPLC. When this determination method was applied to M-glyceride analysis, the standard deviations (SD) were less than 5.5% of the mean values (n = 4). This shows that the accuracy of this method is enough for the determination method of M-glyceride.
